# A Manual of Select Medical Bibliography, in Which the Books Are Arranged Chronologically According to the Subjects, and the Derivations of the Terms and the Nosological and Vernacular Synonyms of the Diseases Are Given. With an Appendix, Containing Lists of the Collected Works of Authors, Systematic Treatises on Medicine, Transactions of Societies, Journals, &c.

**Published:** 1836-04

**Authors:** 


Art. VIII.—*A Manual of Select Medical Bibliography, in which the Books are arranged chronologically according to the Subjects; and the Derivations of the Terms, and the nosological and vernacular Synonyms of the Diseases are given. With an Appendix, containing Lists of the collected Works of Authors, Systematic Treatises on Medicine, Transactions of Societies, Journals, &c. &c. &c.* By John Forbes, m.d., f.r.s.; one of the Editors of the Cyclopædia of Practical Medicine, and of the British and Foreign Medical Review.—Royal 8vo. pp. 404. London, 1835.

It is somewhat singular that there should have appeared two works, during the time over which the first two numbers of our Review may fairly be considered to extend their retrospective jurisdiction, with which the Editors are too intimately connected to render any formal criticism of them admissible into these pages. We, of course, allude to the Cyclopædia of Practical Medicine, and the work of which the title stands at the head of the present notice. Both these works, however, are of too much importance, or, at least,—to avoid every risk of praising ourselves,—*pretend* to be of too much importance, to be overlooked by a Review which professes to notice, not merely all important medical works, but all works published in this country, whether important or not. In the case of the work now before us, still greater delicacy is necessary than in that of the Cyclopædia. *There*, indeed, had we been so minded, without running much risk of compromising our impartiality, we might have selected numerous articles for review and criticism, with which we, as Editors, had nothing to do but to receive them into our collection, and which were of such unquestionable excellence in themselves, and the avowed productions of such distinguished authors, that even the warmest eulogies would have been received from us without cavil or remark. In the present case, however, matters stand on a very different footing; and the utmost that would be allowable on our part, or that could be received on the part of our readers, would be to give a dry statement of the nature and plan of the work, and some specimens of its execution, without commentary or remark. This is what we shall now do; and perhaps the simplest and least offensive way in which our task can be accomplished, is, in the first place, to extract such portions of the Preface as refer to the plan of the work; and, secondly, to insert a few specimens, taken at random from different portions of it.

"The following Bibliographical Collections were originally formed to supply a deficiency in the individual articles of the Cyclopædia of Practical Medicine, and the greater portion of them was published in the Supplement to that work. They are now republished, with additions, with the view of filling, in some degree at least, a very important blank in the medical literature of this country which has long been felt to exist. The plan followed in the Compilation is somewhat different from any with which I am acquainted; and I am in hopes that I have not altogether failed in the object I had in view throughout—of rendering it of easy reference and in every way practically useful. To the attainment of this object I have sacrificed all minor considerations, whether having reference to my own ease and convenience, or to the credit that might attach to the display of such erudition as I am master of, or which the extensive researches which my task imposed upon me might have easily supplied.

"Had I been ambitious of such a display, I might have readily gratified it by the accumulation of references to the miscellaneous writings of physicians in everybody's hands,—to the innumerable monographs which have been printed under the form of Inaugural Dissertations in the various European Universities,—and, lastly, to the individual cases of disease, which form so large a portion of medical periodical literature; and this I might have done with little or no personal research, by merely transferring to my little work—then little no longer—the pages of Haller and Ploucquet, the Bibliographical Notices in the Dictionnaire des Sciences Médicales, and a moiety of the Indexes of the periodical medical collections, now of almost unmanageable size, in this and other countries. But want of space as well as want of inclination, and the governing principle, practical utility, have alike prevented such a consummation; although I am free to confess that so desirable a result has not been attained without the sacrifice of hundreds of pages of manuscript, which had been compiled before what I regard as the proper plan had been thoroughly matured.

"Generally speaking, the reader will find the following pages to consist exclusively of Bibliography, in the stricter sense of the word, that is, an account of *books* only. The sole exceptions to this, of a general kind, will be found to be the references to the great Medical Dictionaries (of which the individual articles have been regarded as distinct works), and to certain Collections of Inaugural Dissertations and Minor Works, which have attained high consideration and a general circulation. Few persons are aware of the very large space in Medical Bibliographies commonly occupied by the titles of Inaugural Dissertations, which have never, properly speaking, been published, and which are not, therefore, accessible to any reader, unless he visits the library of the particular university in which the authors obtained the doctorate. To reproduce, *ad infinitum*, these titular treatises in our bibliographies is not simply to dazzle the ignorant with the semblance of learning, and at no expense, but is to delude the unwary with the bare imagination of a feast which, in all probability, they can never possibly enjoy.

"References to the slighter monographs, and observations and cases in the collected works of authors, or in treatises ostensibly dedicated to other objects, or in the Transactions of Societies and Periodical Journals, are not open to the same objections, and the only reason for excluding them from the following pages is the impossibility of admitting them without superseding others of greater moment, or extending the compilation beyond the prescribed bounds. It will, indeed, be found that the rule of excluding such references has by no means been rigidly adhered to, more particularly where the matter seemed of extraordinary importance, or where the number of works on the particular subject was very limited . . . . It has been after much consideration that the Chronological Order has been adopted in the arrangement of the titles of the different works; this plan appearing to possess important advantages over the alphabetical arrangement, more especially in such a collection as the present, where the list of works is, under no one head, very extensive. The placing the date of the publication in a conspicuous form *before* the title, will, it is hoped, render the advantages of the arrangement still more conspicuous. The adherence to the chronological order has led to one result, which some may think not always satisfactory, that namely, of recording preferably the earliest edition of the respective works. Many exceptions to this will indeed be met with; and, now the work is finished, I regret that, while I prefixed this earliest date to the title, I did not also subjoin the dates of the subsequent editions. The omission, however, will scarcely be found a practical inconvenience, since it is always easier to procure a later than an early edition.

"In a great majority of instances the exact words of the titles have been given, although it has been found necessary, for the sake of economising space, very generally to curtail them: in no case, I trust, will there be found any difficulty of recognizing the work under the title given to it. . . . The Etymology and Synonymy prefixed to the different articles is, I believe, a new feature in works of this kind. I think, however, it will be generally admitted to have a natural relation to the main subject, and, at all events, it cannot fail to be useful to students and the younger members of the profession. The greater number of the modern languages of which synonyms are given, are become, in these days, almost necessary acquisitions for the accomplished physician. If of this number the Dutch, Danish, and Swedish, may be deemed less essential, I shall be less solicitous about the accuracy of the synonyms in these, and less concerned for my own ignorance of them: for the names of diseases in the two last-named languages I am entirely indebted to Nemnich's *Lexicon Nosologicum Polyglotton,* a work of some merit, but far from accurate." (*Pref.*)

The same general plan is followed with each article; the following particulars being given in order: 1. The Derivation of the Term or Terms; 2. The Nosological Synonyms; 3. The Vernacular Synonyms; 4. The titles of the books having reference to the particular disease, organ, or subject, in chronological order.

To avoid all imputation of partiality in our selections, we shall take the initial and final article under the two first letters in the alphabet, introducing merely a few of the titles of the books given under each.

"ABDOMEN, EXPLORATION OF—DISEASES OF.

DERIVATION. The derivation of the word *abdomen* is uncertain. It is commonly said to be formed from the Latin verb *abdo,* to conceal, either simply from this word, (*abdo, abdomen,* as *lego, legumen,*) or from *abdo* and *omentum* conjointly. It does not, however, appear why this particular cavity should be so distinguished, seeing that its contents are not more *hidden* than those of the chest or head.

VERNACULAR SYNONYMS. *Greek,* Γαστηρ, υπογαστριον, υποκοιλιον, υπητριον. *Latin,* Abdomen, venter. *English,* Belly, stomach, paunch. *German,* Unterleib. *Dutch,* Onderbuik. *French,* Ventre, bas ventre. *Italian,* Ventre, pancia, abdomine. *Spanish,* Vientre, barriga, panza.

1696 *Baglivi, G. M.* De observatione hypochondriorum in acutis (Prax. Med. lib. i. c. 6.) *Rom.* 8vo.

1698 *Stahl, G. E.* De vena portæ porta malorum hypochondriaco-splenetico-suffocativo-hæmorrhoidariorum. *Hall.* 4to.

1742 *Furstenau, J. H. A.* Abscessuum musculorum abdominis exempla. *Rintel.*

1749 *Quelmalz, S. T.* Programma de frictionibus abdominis (Haller Disp. ad Morb. vii. 317.) *Lips.* 8vo.

1751 *Kaempf, J.* De infarctu vasorum ventriculi (Baldinger Syll. iii.) *Basil.*

1752 *Koch, D. Æ.* De infarctibus vasorum in infimo ventre (Baldinger Syll. iii.) *Argent.*

1754 *Elvert, F.* Novæ observationes de infarctibus venarum abdominalium (Baldinger Syll. iii.) *Tubing.*

1755 *Faber, G. B. Brotbeck, A. T.* Ulterior expositio novæ methodi Kempfianæ (Baldinger Syll. iii.) *Tub.*

1797 *Corbella.* Tratado sobre las enfermedadas internas y mas agudas del vientre. *Madr.* 8vo.

1803. *Albers, J. F.* Ueber pulsationen im unterleibe. *Brem.* 8vo.

(With 17 additional titles; in all, 27.)

AUSCULTATION.

DERIV. Lat. *auscultatio,* listening, hearkening, from *ausculto,* to listen, from the ancient *auses* pro *aures,* quasi *aures culto,* i. e. *aures colo.*

VERN. SYN. *Gr.* Ακροασις. *Lat.* Auscultatio. *Eng.* Auscultation, auricular exploration, listening. *Ger.* Das anhören, das erforschen durch das gehör. *Fr.* Auscultation. *Ital.* L'ascoltare, ascoltazione. *Span.* Auscultacion, accion de escuchar.

1728 *Lancisi, G. M.* De motu cordis, &c. *Rom.* fol.

1748 *Brendel, J. G.* De motu cordis Lancisiano. *Goett.* 4to.

1761 *Auenbrugger, Leop.* M. D. Inventum novum ex percussione thoracis humani ut signo abstrusos interni pectoris morbos detegendi. *Vindob.* 8vo.

1770 *Roziere, De la Chassagne,* M. D. Manuel des pulmoniques, ou traité complète des maladies de la poitrine: il y a joint une nouvelle méthode, &c. traduite du Latin d'Avenbrugger. *Par.* 8vo.

1808 *Corvisart, J. N.* Nouvelle méthode pour reconnaître les maladies internes de la poitrine, par Avenbrugger (transl.) *Par.* 8vo.

1813 *Dessans,* Essai sur la percussion de la poitrine (Diss. Inaug.) *Par.*

1817 *Double, F. J.* Séméiologie générale (t. ii. p. 31.) *Par.*

1819 *Laennec, R. T. H.* De l'auscultation médiate, ed. i. 1819, ed. ii. 1826, ed. iii. par Mer. Laennec, 3 vol. *Par.* 1831. 8vo. *Id.* translated by Dr. Forbes, 1st ed. *Lond.* 1821, 2d. 1827, 3d. 1829, 4th. 1834.

(With 44 additional articles; in all, 52.)

### BARBIERS.

DERIV. Unknown: vernacular Indian word.

NOS. SYN. *Beriberia:* Sauv. Sag. Cull. *Synclonus beriberia:* Good. *Beriberia Indica:* Bontius. *Beriberi:* Manget. Linn. *Asthenia beriberia:* Young. *Paralysis beriberi:* Tulp.

(For Literature, see PARALYSIS; also CLIMATE.)

### BATHS AND BATHING.

DERIV. The English word *bath* is of Saxon origin, *bad,* which still retains the original form in the modern German.

SYN. (1. *Bath;* 2. *cold bath;* 3. *warm bath.*)

*Gr.* 1. Βαλανειον; 2. ψυχρολουσια; 3. θερμολουσια. *Lat.* 1. Balneum, balnea, balineum, baliniæ; 2. balneum frigidum; 3. balneum calidum. *Eng.* 1. bath; 2. cold bath; 3. hot or warm bath. *Ger.* 1. bad; 2. kaltes bad; 3. warmes bad. *Dut.* 1. bad, 2. koud bad, koel bad; 3. warm bad, heet bad. *Fr.* 1. bain; 2. bain froid; 3. bain chaud. *Ital.* 1. bagno; 2. bagno freddo; 3. bagno caldo. *Span.* 1. baño; 2. baño frio; 3. baño caliente.

1533 Anon. De balneis omnia quæ extant apud Græcos, &c. *Venet.* fol.

1543 *Fumanellus, Ant.* Consilium, &c. et de balneis ferratis et aquæ simplicis. *Basil.* 8vo.

1552 *Clivoli, Bart. A.* De balneorum naturalium viribus. *Lugd.* 4to.

1565 *Guintherus, Joan.* Commentarius de balneis in tribus dialogis. *Argent.*

1568 *Turner, W.* M. D. Book of the nature and properties of the baths of England, &c. *Collen,* fol.

1579 *Rulandus, M.* Balnearium restauratum. *Basil.* 18mo.

1622 *Baccius, And.* De thermis libri septem. *Rom.* fol.

1633 *Jorden, E.* Of bathes and mineral waters. *Lond.* 4to.

1636 *Brancaleonis, J. F.* De balneorum utilitate, ex Hippocrate. *Par.* 8vo.

1641 *Oliva, Chr. de,* Trattado de los baños de aqua dolce. *Saragossa,* fol.

(With 95 additional titles; in all, 105.)

### BRONCHOCELE.

DERIV. From βρογχος, the throat, and κηλη, swelling: βρογχοκηλη.

NOS. SYN. *Bronchocele botium:* Roncalli. *Bronchocèle:* Sauv. Vog. Auct. Var. *Gongrona:* Hipp. Galen, Vog. *Deironcus glandularis, Thyroncus:* Swed. *Tracheocele:* Heister. *Thyrocele, Hernia bronchialis, Hernia gutturalis, Hernia colli:* Auct. *Thyrophraxia:* Alibert. *Cynanche thyroidea:* Conradi. *Struma:* J. Frank, Auct. Var. *Trachelophyma:* Sagar. *Botium:* Paré. *Thyreophyma:* J. P. Frank.

VERN. SYN. *Gr.* Γογγρωνη, βρογχοκηλη. *Lat.* Guttur tumidum, guttur globosum. *Eng.* Swelled neck, Derbyshire-neck, Derby-neck. *Ger.* Kropf, windkropf, luftröhrenbruch, dicker hals. *Dut.* Gorgelgezwel, krop, kropgezwel. *Dan.* Keigbyld. *Swed.* Struma. *Pol.* Wola. *Fr.* Goître, gouëtre. (Sauv.) *Ital.* Gozzo, gozzaja, broncocele. *Span.* Papera, bocio, seca, lamparones.

1752 *Astruc, J.* M.D. Traité des tumeurs, 2 vol. *Par.* 12mo.
1759 *Astruc, J.* M.D. Recueil concernant le traité des tumeurs. *Par.* 12mo.
1762 *Prosser, Thos.* An account and method of cure of the bronchocele or Derby neck. *Lond.* 8vo.
1777 *Read, M.* Mémoire sur les bronchocèles du pays Messin. *Nancy,* 12mo.
1779 *Wilmer, B.* Cases in surgery, with the method of curing the bronchocele. *Lond.* 8vo.
1782 *Prosser, Th.* On bronchocele and ovarian dropsy. *Lond.* 4to.
1787 *Valentin, J. L.* Dissertatio de struma bronchocele dicta. *Nancy,* 4to.
(With 43 additional titles; in all 50.)"

The Appendix comprises an extensive selection of works arranged under the following heads:—

1. The works of the principal writers on medicine which have been published in a collected form.
2. Systematic treatises on the whole or the greater part of practical medicine, by single authors.
3. Miscellaneous observations and memoirs on different parts of practical medicine, by single authors.
4. Collections of inaugural dissertations, and of the minor works of different authors, republished in a distinct form.
5. Transactions, memoirs, &c. published by colleges, academies, societies, &c.
6. Periodical works, or works published at periods more or less regular, not the productions of colleges, academies, or societies.

